# Interplay between endocannabinoids and dopamine in the basal ganglia: implications for pain in Parkinson’s disease

**DOI:** 10.1186/s44158-024-00169-z

**Published:** 2024-05-14

**Authors:** Maria Mancini, Alessandra Calculli, Deborah Di Martino, Antonio Pisani

**Affiliations:** 1 Department of Brain and Behavioral Sciences, University of Pavia, c/o Mondino Foundation Via Mondino, 2, Pavia, 27100 Italy; 2 IRCCS Mondino Foundation, Pavia, 27100 Italy

**Keywords:** Dopamine, Endocannabinoids, Parkinson’s disease, Pain, Ion channels

## Abstract

Pain is a complex phenomenon, and basal ganglia circuitry integrates many aspects of pain including motor, emotional, autonomic, and cognitive responses. Perturbations in dopamine (DA) signaling are implicated in the pathogenesis of chronic pain due to its involvement in both pain perception and relief. Several lines of evidence support the role of endocannabinoids (eCBs) in the regulation of many electrical and chemical aspects of DAergic neuron function including excitability, synaptic transmission, integration, and plasticity. However, eCBs play an even more intricate and intimate relationship with DA, as indicated by the adaptive changes in the eCB system following DA depletion. Although the precise mechanisms underlying DA control on pain are not fully understood, given the high correlation of eCB and DAergic system, it is conceivable that eCBs may be part of these mechanisms.

In this brief survey, we describe the reciprocal regulation of eCB-DA neurotransmission with a particular emphasis on the actions of eCBs on ionic and synaptic signaling in DAergic neurons mediated by CB receptors or independent on them. Furthermore, we analyze the eCB-DA imbalance which characterizes pain condition and report the implications of reduced DA levels for pain in Parkinson’s disease. Lastly, we discuss the potential of the eCB-DA system in the development of future therapeutic strategies for the treatment of pain.

## Introduction

The identification of the cannabinoids receptors (CB-Rs) in the early 1990s [1, 2] suggested that they serve not merely as binding sites for psychotropic substances extracted from *Cannabis sativa* plant flowers (cannabinoids) but rather function as mediators of endogenous signaling molecules, physiologically operating as messengers in the mammalian central nervous system (CNS). This discovery prompted the search for endogenous agonists, or endocannabinoids (eCBs), which mimic the psychomotor effects of cannabinoids [3].

Since then, the eCB system has been demonstrated to play important physiological roles in the control of emotional responses, cognition, feeding, pain, motivated behaviors, and motor control. However, the precise mechanisms employed by eCBs to mediate their effects remain partially understood, due to the multiplicity and complexity of their actions. In fact, both CB1 and CB2 are G protein-coupled receptors (GPCRs), which influence the biochemical and electrical state of a cell through complex mechanisms involving kinases, phosphatases, transcription factors, and ion channels. Moreover, the interaction with other systems of neurotransmitters and the modulation of their activity can induce changes in synaptic transmission, promoting long-term changes in synapse efficacy and modifying the structural organization of neuronal circuitries.

CB-Rs are widely distributed throughout the CNS. Of note, their robust expression within the basal ganglia circuitry has stimulated investigation on the possible interplay between eCBs and dopamine (DA) system, not only in the context of movement disorders but also in pain research. Several preclinical and clinical studies have demonstrated that eCBs are crucial modulators of DAergic transmission [4], and that DA agonists may have an antinociceptive effect [5]. Conversely, DA depletion leads to enhanced nociceptive responses [6–11] and induces adaptive changes in the eCB system [12–15]. Accordingly, Parkinson’s disease (PD) patients report comorbid pain conditions and higher pain rating [16, 17].

Despite such evidence, the specific mechanisms through which intracellular effectors recruited by eCBs directly or indirectly interact with DA-dependent signaling effectors to affect pain perception have been only partially identified. However, a review of all the potential cellular and molecular mechanisms is beyond the scope of this paper, and we refer the reader to a number of excellent comprehensive reviews (see [18–22]). Our brief survey will rather focus on the available evidence on the interactions between eCBs and DA. In PD, DA depletion provides an ideal clue to review and discuss the compensatory changes of the eCB system, as a potential mechanism contributing to pain in PD. Advancing our knowledge on these basic mechanisms might reveal potential molecular targets for drug development in PD-related pain.

## Endocannabinoid-dopamine crosstalk

### eCB and CB-Rs in the basal ganglia

**Fig. 1:**
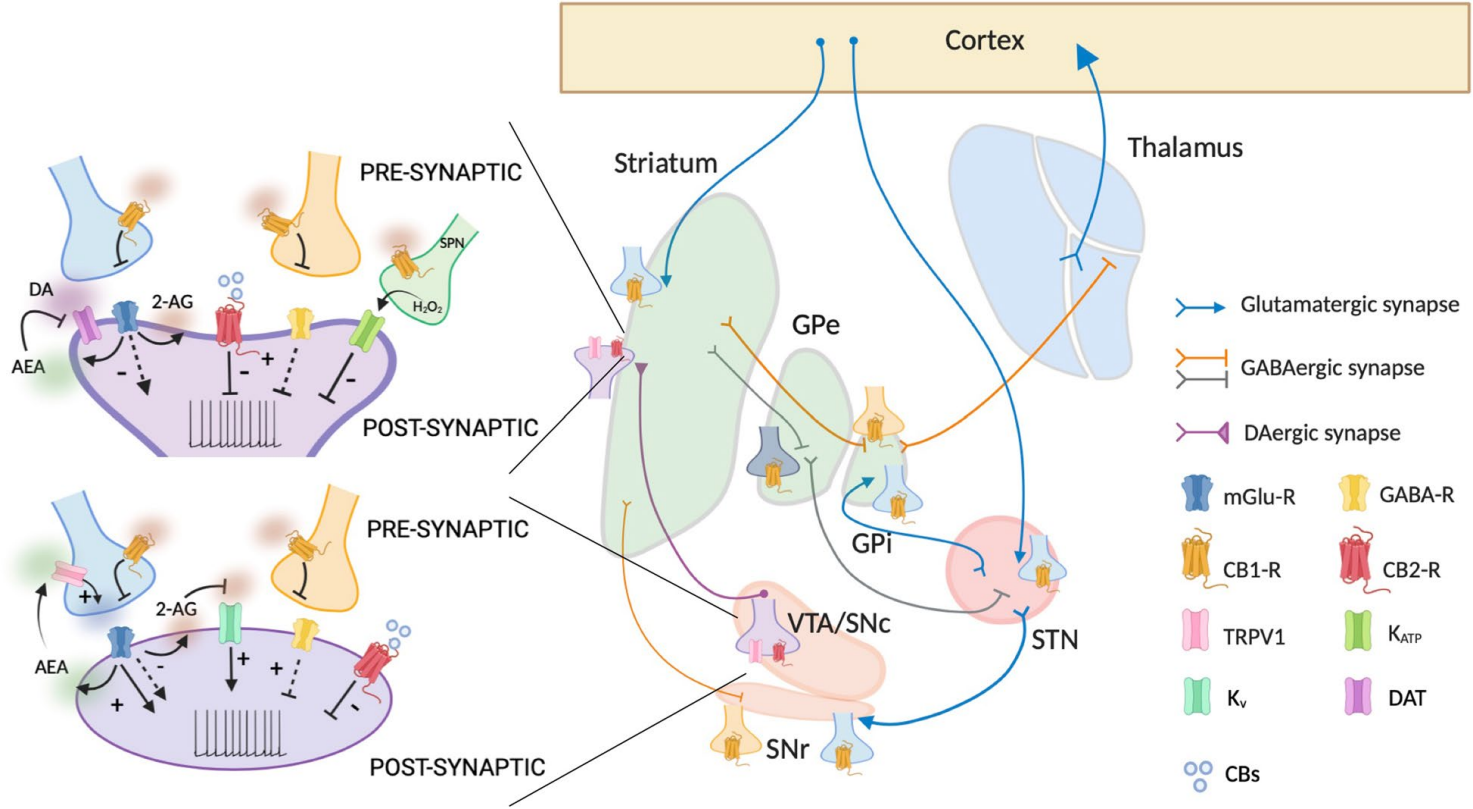
Distribution of CB-Rs within the basal ganglia circuitry and eCB/CB-dependent modulation of DA release via direct and indirect mechanisms. eCBs can control both excitatory and inhibitory neurotransmitter release and indirectly modulate axonal and somatodendritic DA release. A direct action of eCBs on DA axons and cell bodies occurs following binding to CB2-R or K^+^ channels which results in inhibition or increase in DA release

The presence of eCB-Rs within the basal ganglia circuitry has been demonstrated by several neuroanatomical studies. The expression of the CB1-R has been found in the glutamatergic corticostriatal afferences in both dorsal and ventral *striatum* [23, 24] and the projections of striatal GABAergic neurons innervating the *globus pallidus* (GPi and GPe) and the *substantia nigra pars reticulata* (SNpr) [25, 26]. Some studies have also confirmed the distribution of CB1-R in the glutamatergic terminals innervating the *subthalamic nucleus* (STN) and the GPi/SNpr [27] (Fig. 1).

Also, CB2-R was found in the *striatum*, GP, DAergic neurons of the *ventral tegmental area* (VTA) and SN, and in the basal *thalamus* [28–32] (Fig. 1).

In addition to the classical CB-Rs, other putative eCB receptors exist. Among these, the transient receptor potential vanilloid type-1 (TRPV1) channel can be activated by several exogenous and endogenous stimuli. TRPV-1 has been identified in both the axonal terminals and the soma of nigrostriatal DAergic neurons [33–35] (Fig. 1).

CB-Rs and the putative receptors are activated by eicosanoids such as N-arachidonoyl-ethanolamide (AEA) or *anandamide*, and 2-arachidonoyl-glicerol (2-AG), and by cannabinoids and their analogues.

**Fig. 2:**
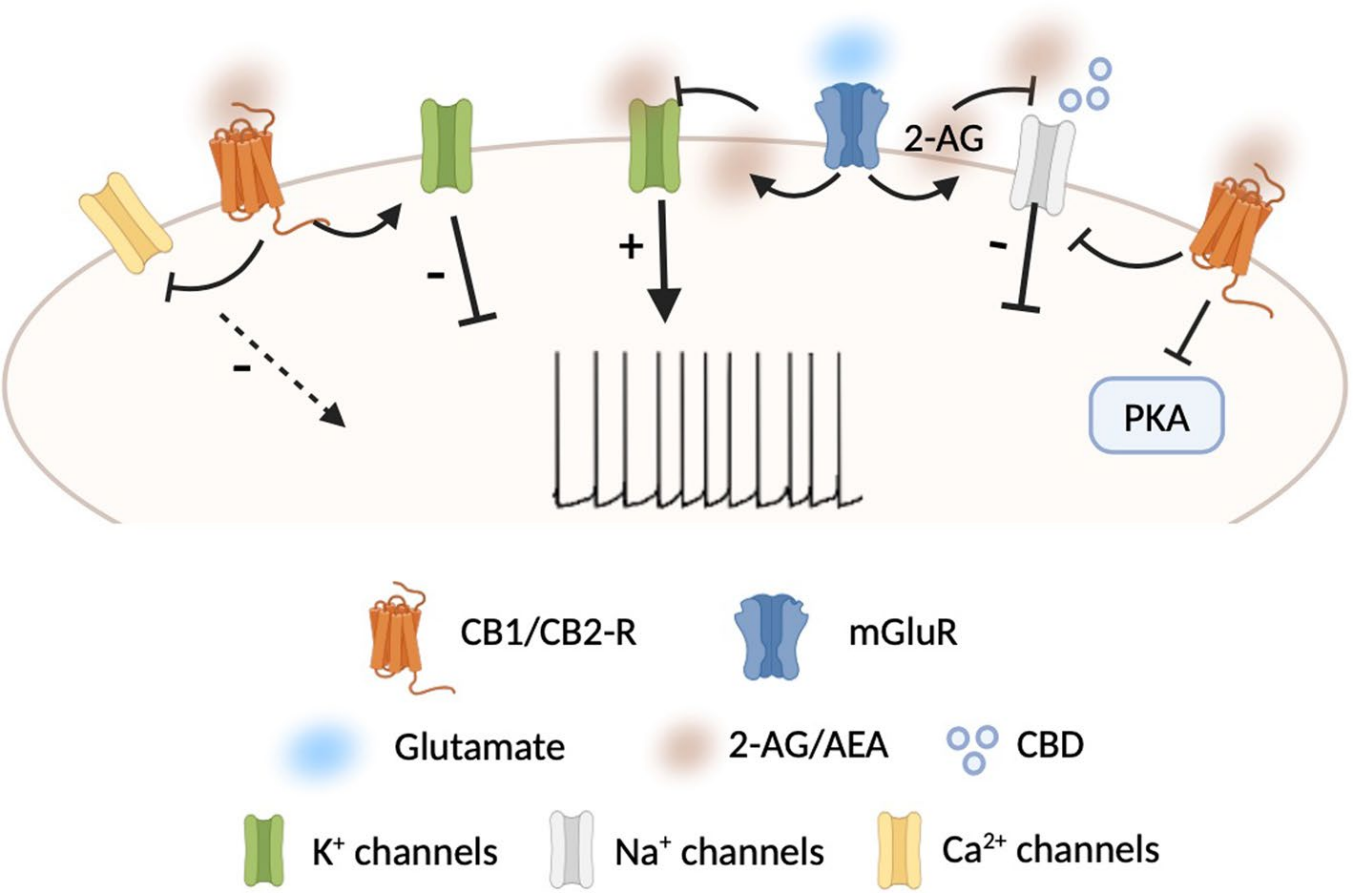
Schematic representation of eCB- and CB-mediated changes in neuronal excitability via action at ion channel level. The release of eCBs causes activation or inhibition of several classes of ion channels. In some cases, the modulation of the ion channels by eCBs (or CBs) is consequent to the activation of CB-Rs, whereas in others, the action of eCBs on these channels is direct or occurs independently of known CB-Rs

As GPCRs, both CB1-R and CB2-R activate heterotrimeric G proteins and use similar transduction systems. CB1-R is coupled to Gα_i_ and Gα_o_ proteins which inhibit protein kinase A (PKA). PKA mediates most of the effects of CB1-R by phosphorylating and regulating the function of a wide array of cellular substrates such as voltage-gated K^+^, Na^+^, and Ca^2+^ channels (Fig. 2), ionotropic glutamate, and GABA receptors and transcription factors. Noteworthy, the modulation of ion channels following CB1-R activation can either depend on its coupling with Gα_olf_ protein or be independent of cAMP/PKA signaling. A good example is the gating of G protein-coupled inwardly rectifying K^+^ channels (GIRKs) at presynaptic level in the *nucleus accumbens* (NAc) [36]. Also, CB1-R can modulate intracellular Ca^2+^ levels and regulate ligand- and voltage-gated ion channels through the liberation of the Gβγ subunit upon receptor activation.

CB1-R is mainly expressed in presynaptic terminals in homo- and heterodimeric structures; however, it can also be present at postsynaptic level where it can form heterodimers with other GPCRs including DA receptors [37–39]. Depending on the DA-R bound, CB1-R can contribute in different ways to the modulation of both excitatory and inhibitory synaptic functions as well as to the decrease of neurotransmitter release in the basal ganglia circuitry (for rev., see [40]).

Similar to CB1-R, CB2-R is coupled to Gα_i_ and Gα_o_ proteins and its stimulation results in PKA inhibition. Although its role in the CNS is still controversial, some studies have shown its involvement in modulation of ion channels and DA levels [41–44].

CB1-Rs and CB2-Rs can also activate other intracellular signaling cascades, including the mitogen-activated protein kinase (MAPK) cascade, the phosphatidylinositol 3-kinase (PI3K) cascade, and nitric oxide production [45].

TRPV1 channels are characterized by weak voltage sensitivity and nonselective permeability to monovalent and divalent cations including Mg^2+^, Ca^2+^, and Na^+^. Their activation has been shown to modulate synaptic transmission and neurotransmitter release [46–48].

### Dual regulation of DA/eCB neurotransmission

DA neurons are fine-tuned by eCBs, which can regulate DA neurotransmission at midbrain cell bodies, at DA axon terminal endings or by acting on DA neuron effector sites in the striatum (Fig. 1).

The modulation of DA release by eCBs in midbrain neurons occurs via different mechanisms, according to the target receptor activated.

DA neurons in midbrain do not express CB1-Rs [49]; however, many other neuronal populations connected with DAergic cells present CB1-R and are modulated by eCBs. Thus, eCBs indirectly control DA cells through modulation of local circuitry, facilitating or suppressing DAergic neuron activity [40, 50, 51], primarily through 2-AG (Fig. 1).

In contrast to CB1-Rs which are not expressed by midbrain DAergic neurons, CB2-Rs are present on cell bodies [29], and their activation by exogenous agonists inhibits DAergic neuronal firing [52, 53] (Fig. 1); however, whether endogenous molecules driving CB2-R signaling act in a similar way to reduce DA release has not been established yet.

In the SN, an increase in the excitatory neurotransmission through a tonic modulation of the glutamatergic synaptic inputs onto DAergic neurons by the eCB AEA has been measured. Such enhancement occurs after activation of TRPV1, and the effect of AEA on DAergic transmission results in an increase in DA levels [46, 47] (Fig. 1). Of note, the modulation of DA release by AEA occurs also in response to peripheral noxious stimulation in freely moving animals [47]. While TRPV1 channels have been found also on DAergic cells, it is unclear whether their activation has a direct action at postsynaptic level.

At striatal level, DA neurotransmission is modulated locally at presynaptic axonal release sites by several afferent inputs (i.e., cholinergic and glutamatergic inputs). In such context, also eCBs participate in the local modulation of axonal DA release. Specifically, it has been observed that CB1-R activation inhibits the evoked release of DA [54] (Fig. 1). The exact cellular localization of CB1-Rs responsible for such inhibition is not clear yet; however, polysynaptic and indirect mechanisms have been implicated in the decrease of DA levels.

Interestingly, also CB2-R activation on DA terminals seems responsible for causing inhibition of striatal DA release [55]. This phenomenon has been observed following exogenous activation; whether endogenous agonists do the same influencing DA tone and DA-dependent behavior is not established yet (Fig. 1).

Finally, the localization of TRPV1 channels at striatal level [34] implicates a possible involvement of these receptors in the modulation of the DA tone. These channels have already been demonstrated to be involved in the control of GABA synapses and in the regulation of excitatory transmission [48, 56], thus, it is plausible that their activation indirectly may impact DAergic transmission.

The regulation of DA neurotransmission by eCBs at striatal sites occurs also at the level of the regulatory mechanisms that control the concentration of DA at synaptic cleft. Specifically, AEA increases extracellular DA levels [57] via inhibition of DA transporter (DAT) activity [58] (Fig. 1). The inhibition of DA uptake by eCBs and other cannabinoids has been observed in native tissue but also in heterologous expression systems [59–61]. Collectively, these data clearly show that eCBs control DA neurotransmission at multiple sites of action within the basal ganglia.

The mutual interaction between eCBs and DA is not only dependent on the ability of eCBs to modulate DA release but also vice versa. Indeed, DAergic neurons can themselves produce eCBs from their somata and dendrites [62], thereby modulating the retrograde signaling. The release of eCBs from DAergic neurons is consequent to an increase in the firing of DAergic cells which causes the mobilization of 2-AG (Fig. 1). Moreover, it has been observed that DA, following binding to D2-R, can promote the postsynaptic release of eCBs from striatal spiny projection neurons (SPNs) which, in turn, influence transmitter exocytosis through the activation of presynaptic GPCRs [63].

In general, the stimulation of D2-Rs by DA causes increases of striatal eCB levels due to an increased synthesis and an inhibited degradation [64, 65]. Interestingly, D2-R and CB1-R are co-expressed, and CB1-R activation by AEA can enhance the effects of D2-R activation [66, 67], thereby amplifying e-CB production.

### eCB action independent of CB-Rs: focus on ion channels

Endogenous cannabinoids, as well as exogenous and synthetic congeners, have been shown to target other molecular substrates in addition to the classical CB-Rs. In particular, these molecules can modify the gating of several classes of ion channels and affect neuronal excitability and neurotransmitter release.

In fact, both eCBs and cannabidiol (CBD), a phytocannabinoid extracted from *cannabis sativa*, are able to interact with voltage-gated sodium channels (Na_v_). Specifically, 2-AG is able to induce channel block and inhibit Na^+^ currents or induce changes in biophysical properties of activation/inactivation of Na_v_ channels, thereby affecting their function [68] (Fig. 2). Similarly, CBD has inhibitory effects on Na^+^ conductance without specificity to any of Na_v_ subfamily [69–73] and can directly interact with Na_v_ channels to inhibit their activity [74, 75] (Fig. 2). Interestingly, CBD binds the channel in more than a single site, and the binding sites are not positioned to physically occlude the pore. Moreover, the inhibition is allosteric and determines a stabilization of the inactivated state.

In addition to Na_v_ channels, cannabinoids have been reported to interact also with K^+^ channels decreasing the peak currents [76]. In this regard, it has been shown that the inhibition of native A-type K^+^ channels by 2-AG in DAergic neurons causes an increase in their excitability, inducing a transition from tonic firing to burst discharge and thus affecting DA release [77] (Fig. 2).

With these results in mind, and considering the close relationship between cannabinoids and DAergic system, it can be speculated that CBD and eCBs could exert their analgesic effects, at least in part, through a direct action on ion channels whose modulation causes changes in neuronal excitability and in DA release.

## Endocannabinoid-DA interaction: pathological imbalance and implications for pain

### eCB-DA imbalance in PD patients

Clinical studies support the evidence for a reciprocal interplay between DA and eCBs. In individuals with PD, the levels of eCBs measured in the cerebrospinal fluid (CSF) were significantly higher as compared to control subjects. This finding was observed either in drug-naive, recently diagnosed subjects, or in patients with a longer disease duration, undergoing drug washout [15]. Of interest, eCBs levels did not differ in these two subgroups of patients, indicating that elevations of CSF eCBs are independent of disease duration. However, in patients undergoing DAergic treatment, a normal eCB content was measured, not different from controls, suggesting that an adaptive, compensatory mechanism occurs during DA denervation. Evidence for a similar, adaptive change emerges from binding studies performed in post-mortem samples from PD patients. Measurement of CB1-R binding and its coupling to G proteins revealed higher stimulation by CB1 receptor agonists of [35S] GTPcS binding both in the caudate and putamen, along with an enhanced CB1 receptor binding [78]. Overall, the reported changes in eCB levels with or without DA replacement suggest that the DA/eCB interplay is highly dynamic.

The general view of this dual interaction is further confirmed by additional clinical hints and supported by preclinical findings (see below). Indeed, while DA supplementation is known to improve dramatically the motor performance observed in hypokinetic disorders, such as PD, the therapeutic use of CBs has been approved for hyperkinetic disorders, such as Tourette’s syndrome and other tic disorders [79, 80], further supporting the notion of a dual interplay between DA and eCB system.

### eCB-DA imbalance in models of PD

The close interaction between DA and eCBs is clearly evident not only in PD patients but also in experimental PD models. In fact, in multiple toxin-induced rodent and nonhuman primate PD models, as well as in genetic rodent PD models, eCB system is reorganized with changes measurable at level of concentration, clearance, and activity.

In particular, in 6-OHDA-denervated rats and in reserpine-treated rats, enhanced levels of eCBs have been measured in the basal ganglia circuitry [12, 81]. However, the observed enhancement is a consequence of a decreased cleavage and not dependent on an increased synthesis [12]. In line with rodent data, both AEA and 2-AG levels were increased in the caudate of non-human primates treated with MPTP [82].

In contrast to toxin-induced PD models, eCB levels were unchanged in a genetic mouse model of PD carrying the recessive mutation in the PTEN-induced putative kinase 1 (PINK1) gene responsible for early-onset PD [83]. However, in spite of unaltered eCB levels, the synaptic responses to CB1-R activation were impaired, and a significant decrease of binding capacity of CB1R agonists was found in PINK1 knockout mice, again supporting the notion of a mutual interplay between DA and eCBs.

Although some differences exist among the various PD experimental models regarding the level of eCBs altered following DA depletion, the changes observed in the eCB system were in all the models reverted after DAergic therapy with levodopa or a D2-R agonist [13, 15, 82, 83] suggesting that the upregulation of the eCB system consequent to DA depletion is an adaptive, dynamic homeostatic mechanism aimed at compensating the effects of DA loss in the basal ganglia.

Such interplay is further corroborated by a number of preclinical findings. CB-R agonists potentiated reserpine-induced hypokinesia [84] and DA receptor antagonist-induced catalepsy [85]. Conversely, they were able to reduce quinpirole-induced hyperlocomotion [86] and amphetamine-induced hyperactivity (for rev., see [87]).

### Effects of soluble products of inflammation on eCB-DA signaling in PD

Neuroinflammation is a well-established feature of PD, and a growing body of evidence suggests that both peripheral and central immune dysregulation can upregulate cytokines such as IL-1β, IL-6, and TNF-α [88], which participate in the development and persistence of pain states during PD. The increased levels of these cytokines in PD patients’ serum and CSF can affect different neural networks and influence synaptic transmission with significant changes in eCB-DA signaling as well as behavioral implications. Intriguingly, a modulatory effect on both excitatory and inhibitory neurotransmissions at striatal level has been reported for IL-1β with involvement of eCB and DAergic systems. As a matter of fact, the exposure to this cytokine causes enhancement of spontaneous excitatory currents via the activation of presynaptic TRPV1 [89] and a reduced sensitivity of CB1-R [90]. In addition, IL-1β reduces GABAergic inhibition inducing SPN hyperactivation [91]. Analogously, TRPV1 and CB1-R seem to be implicated in such inhibitory modulation [92, 93]. The striatal excitatory/inhibitory imbalance induced by IL-1β is coupled to an alteration of DAergic transmission as suggested by the finding that evoked axonal DA release is reduced when IL-1β is upregulated and restored following administration of IL-1 receptor antagonist (IL-1ra) [94]. Moreover, IL-1ra application also rescues striatal CB1-R sensitivity and DA-dependent behavior [95]. Although not exhaustive, these observations clearly confirm the strong connection between eCB and DAergic system and their sensitivity to inflammatory mediators suggesting a contribution for these molecules in causing the eCB-DA imbalance. In addition to IL-1β, other pro-inflammatory cytokines have been proven to affect DA neurotransmission [96]; however, whether this impairment also exerts influence on eCB system and has implications for pain process is still unknown and deserves to be investigated.

### eCB-DA imbalance during pain

The role of DA in pain can be better appreciated in conditions of DA loss such as in PD. Indeed, compelling evidence indicates that up to 80% of individuals with PD manifest pain during the disease course [17]. The degeneration of nigrostriatal DA pathway in PD patients causes hyperalgesia [97], and the administration of levodopa induces an increase in the pain threshold [98, 99]. Intriguingly, toxin-induced rodent models of PD (i.e., MPTP and 6-OHDA) have provided a valuable contribution in understanding the role of DA in pain process. Similar to humans, DA-depleted animals show an increased nociception and decreased nociceptive threshold [10, 100–104] suggesting that the loss of DA in the basal ganglia is involved in the reduction of pain threshold [105]. Of note, the elevation of striatal DA following electrical stimulation of the SN or the direct striatal administration of apomorphine, a D1/D2-R agonist, induces pain inhibition [106]. Similarly, systemic administration of DA agonists determines hypoalgesia by modulating D2 receptors in dorsolateral striatum [107]. In spite of such evidence, both in humans and in models of PD, replacement with DA therapy does not promote recovery from pain, thereby suggesting that other neurotransmitters may play a role in the pain signaling process.

Although the great therapeutic potential of eCB system in the modulation of pain has been widely recognized [108, 109], the involvement of DA/eCB system within basal ganglia circuitry in chronic pain has been less extensively explored [110]. eCBs are elevated at various sites in nociceptive pathways in chronic pain [19] highlighting their role as endogenous analgesics. Based on the existence of a concurrent decrease in DA concentrations observed during pain, the increase in eCB levels might compensate DA decrease. The imbalance in eCB-DA system during pain condition is somehow reminiscent of the alteration observed during PD. However, eCB effects on nociception and pain following DA depletion have been scarcely investigated. Also, whether DAergic drugs are able to restore resting levels of eCBs as in animal models of PD [13] needs to be clarified.

Recently, Nascimento and colleagues [111] analyzed the effect of CBD on nociception in experimental PD, highlighting the ability of this drug to exert antinociception via activation of CB1-R. Moreover, the authors suggest that the analgesic effect induced by CBD is also dependent on an increase in AEA which likely potentiates the activation of CB1. An elevation in AEA levels has been found in 6-OHDA-denervated animals and is dependent on a decreased cleavage [12]; thus, it is plausible that this drug may cause an increase in DA levels acting at the same sites used by eCBs to directly modulate somatodendritic or axonal DA release in the basal ganglia.

## Conclusions

Although the role of DA in pain perception in PD has not been fully elucidated, it is now evident that an altered DA neurotransmission correlates with impaired eCB levels. Hence, the eCB system would represent, in principle, a promising target for the treatment of pain in PD. Additional work is needed to clarify the functional meaning of these interactions, in order to gain a better understanding of the mechanisms through which eCBs affect DA neurotransmission, which may translate into novel opportunities for improving pain treatment.

## Data Availability

No datasets were generated or analysed during the current study.
